# Supplementary material for: Projections of coral cover and habitat change on turbid reefs under future sea-level rise
Source: Proc Biol Sci. 2020 Jun 17;287(1929):20200541. doi: 10.1098/rspb.2020.0541 (PMC7329041; doi:10.1098/rspb.2020.0541)
Supplement: Supplementary Materials [file rspb20200541supp1.docx]

**Projections of coral cover and habitat change on turbid reefs under future sea level rise**

Kyle M. Morgan, Chris T. Perry, Rudy Arthur, Hywel T. P. Williams, Scott G. Smithers

**Supplementary Materials**

**Figure S1.** Location of Paluma Shoals reef complex (PSRC) within Halifax Bay on the central Great Barrier Reef, Australia. Insert shows the configuration of the nearshore turbid reefs that comprise PSRC; shore-attached Paluma Shoals (PS) North and South, and shore-detached Offshore Paluma Shoals (OPS) A, B, C and D.

**Table S1:** Average (± sd) rates of vertical reef accretion (mm y^-1^) at 0.5 m depth intervals. Data is derived from 31 reef cores collected at Paluma Shoals Reef Complex. Depths are relative to lowest astronomical tide datum (LAT). Data taken from Morgan et al., 2016a

**Table S2**. Summary of reef habitat types observed across Paluma Shoals reef complex. Data of benthic cover (%) and mean accretion rates (mm y^-1^) taken from Morgan et al., 2016a and 2016b, respectively. For a description of *Goniastrea* reef flat habitat (H1) see Palmer et al., 2010.

**Table S3.** Modelled estimates of total reef area (grid cells/hectares) under static sea level. Reef geomorphology is simulated backwards and forwards (± 500 year) from present day. The 5% and 95% confidence intervals associated with the modelled mean are shown.


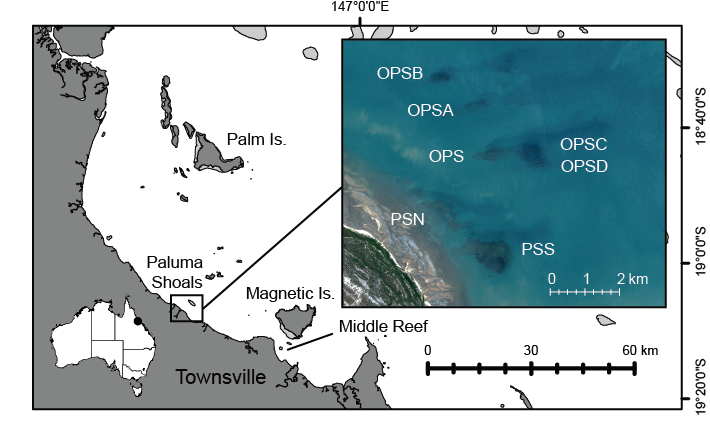


**Figure S1.** Location of Paluma Shoals reef complex (PSRC) within Halifax Bay on the central Great Barrier Reef, Australia. Insert shows the configuration of the nearshore turbid reefs that comprise PSRC; shore-attached Paluma Shoals (PS) North and South, and shore-detached Offshore Paluma Shoals (OPS) A, B, C and D.

**Table S1:** Average (± sd) rates of vertical reef accretion (mm y^-1^) at 0.5 m depth intervals. Data is derived from 31 reef cores collected at Paluma Shoals Reef Complex. Depths are relative to lowest astronomical tide datum (LAT). Data taken from Morgan et al., 2016a

| **Depth (cm below LAT)** | **Reef accretion rate (mm y^-1^)** |  |
| --- | --- | --- |
| > 0 | 1.4 ± 1.1 | above LAT |
| 0-50 | 3.4 ± 4.5 |  |
| 50-100 | 3.8 ± 3.8 | below LAT |
| 100-150 | 2.5 ± 1.3 |  |
| 150-200 | 5.1 ± 4 |  |
| 200-250 | 6.9 ± 9.4 |  |
| 250-300 | 6.4 ± 7.8 |  |
| 300-350 | 5.5 ± 5.2 |  |
| 350-400 | 1.4 ± 0 |  |
| 400-450 | 0 ± 0 |  |

1. Morgan, K.M., Perry, C.T., Smithers, S.G., Johnson, J.A., Gulliver, P., 2016a. Transitions in coral reef accretion rates linked to intrinsic ecological shifts on turbid-zone nearshore reefs 995–998. <https://doi.org/10.1130/G38610.1>

**Table S2**. Summary of reef habitat types observed across Paluma Shoals reef complex. Data of benthic cover (%) and mean accretion rates (mm y^-1^) taken from Morgan et al., 2016a and 2016b, respectively. For a description of *Goniastrea* reef flat habitat (H1) see Palmer et al., 2010.

|  | **Habitat type** | | | | | |
| --- | --- | --- | --- | --- | --- | --- |
|  | H2: Rubble & encrusting coral | H3: *Montipora* & *Acropora* framework | H4: Massive *Porites* & sand | H5: *Turbinaria* carpets | H6: Rubble & sediment-tolerant coral | H7: Sand/mud |
| Depth range (m LAT) | 0-0.5 | 0.5-1.5 | 1.5-2 | 2-3 | 3-4 | > 4 |
| Mean accretion rate (mm y^-1^) | 1.4 | 3.4 | 4.2 | 3.7 | 2.4 | 0.0 |
| Median rugosity (1 - 5) | 3 | 4 | 1 | 5 | 2 | 1 |
| **Benthic cover (%)** |  |  |  |  |  |  |
| Coral | 13 ± 10.2 | 46.7 ± 19.5 | 21.2 ± 11.3 | 71.9 ± 19.7 | 11 ± 9.9 | 1.9 ± 3.8 |
| Sand | 3.3 ± 6.6 | 3 ± 5.8 | 46.8 ± 9.2 | 18.3 ± 18.6 | 28.6 ± 12.9 | 91.1 ± 13.3 |
| Rubble | 74 ± 12.3 | 32.4 ± 16.7 | 17.6 ± 6.7 | 5.4 ± 4.3 | 54.6 ± 12.2 | 4.3 ± 7.8 |
| Dead coral | 5.9 ± 6.2 | 11.5 ± 8.3 | 8.4 ± 9.6 | 4.1 ± 4.8 | 3.5 ± 5.7 | 0.9 ± 3 |
| Macroalgae | 2.5 ± 3.9 | 4.1 ± 7.2 | 0 ± 0 | 0 ± 0 | 0.3 ± 1.6 | 0.1 ± 0.8 |
| **Relative abundance of coral genera (%)** |  |  |  |  |  |  |
| *Acropora* | 19.2 ± 22.7 | 15.7 ± 15.9 | 2.2 ± 3.5 | 3.2 ± 3.4 | 6.4 ± 8.4 | 0.2 ± 1.5 |
| *Turbinaria* | 2.7 ± 5.1 | 7.1 ± 11.2 | 2 ± 5.3 | 79.5 ± 15 | 3 ± 5.3 | 1.3 ± 4.5 |
| *Montipora* | 12.9 ± 11.2 | 54.8 ± 18.8 | 13.4 ± 9.5 | 5 ± 6.2 | 8.3 ± 8.9 | 1.5 ± 4.3 |
| *Porites* | 2.2 ± 4.3 | 1.8 ± 5 | 25.5 ± 15.4 | 1 ± 3 | 7.4 ± 11.2 | 1.7 ± 4.5 |
| Other | 3 ± 4.4 | 3.3 ± 5.6 | 3 ± 4.7 | 1.1 ± 3 | 7.5 ± 12.9 | 1.6 ± 4.4 |

1. Morgan, K.M., Perry, C.T., Smithers, S.G., Johnson, J.A., Gulliver, P., 2016a. Transitions in coral reef accretion rates linked to intrinsic ecological shifts on turbid-zone nearshore reefs 995–998. <https://doi.org/10.1130/G38610.1>
2. Morgan, K.M., Perry, C.T., Smithers, S.G., Johnson, J.A., Daniell, J.J., 2016b. Evidence of extensive reef development and high coral cover in nearshore environments: implications for understanding coral adaptation in turbid settings. Sci. Rep. 6, 29616. <https://doi.org/10.1038/srep29616>
3. Palmer, S.E., Perry, C.T., Smithers, S.G., Gulliver, P., 2010. Internal structure and accretionary history of a nearshore, turbid-zone coral reef: Paluma Shoals, central Great Barrier Reef, Australia. Mar. Geol. 276, 14–29. <https://doi.org/10.1016/j.margeo.2010.07.002>

**Table S3.** Modelled estimates of total reef area (grid cells/hectares) under static sea level. Reef geomorphology is simulated backwards and forwards (± 500 year) from present day. The 5% and 95% confidence intervals associated with the modelled mean are shown.

| **Time (years before/after present day)** | **5% CI** | **Mean number of grid cells (hectares; ha)** | **95% CI** |
| --- | --- | --- | --- |
| -500 | 909 | 1337 (53 ha) | 1669 |
| -400 | 1392 | 1800 (72 ha) | 2147 |
| -300 | 2035 | 2382 (95 ha) | 2743 |
| -200 | 2778 | 3100 (124 ha) | 3485 |
| -100 | 3635 | 4012 (160 ha) | 4440 |
| Present day | 5343 | 5343 (214 ha) | 5343 |
| +100 | 6310 | 6390 (256 ha) | 6469 |
| +200 | 7328 | 7423 (297 ha) | 7521 |
| +300 | 8308 | 8394 (336 ha) | 8480 |
| +400 | 9345 | 9427 (377 ha) | 9521 |
| +500 | 10364 | 10467 (419 ha) | 10581 |
